# Regulation of mitochondrial function by PINK1, Parkin and DJ-1

**DOI:** 10.1186/1750-1326-8-S1-O13

**Published:** 2013-09-13

**Authors:** Emilie Giaime, Jie Shen

**Affiliations:** 1 Center for Neurologic Diseases, Department of Neurology, Brigham & Women’s Hospital, Program in Neuroscience, Harvard Medical School, Boston, MA, USA

## 

Loss-of-function mutations in PTEN-induced kinase 1 (PINK1), Parkin and DJ-1 genes have been linked to recessively inherited forms of Parkinsonism. PINK1 is a putative serine-threonine kinase containing a mitochondrial localization signal, whereas Parkin and DJ-1 function as E3 ubiquitin-protein ligase and scavenger for reactive oxygen species (ROS), respectively. Interestingly, all three of them have been implicated in the control of the mitochondrial function. In this study using primary mouse embryonic fibroblasts (MEFs), primary neurons and mice brain, we investigated how the mitochondrial respiration is affected by the loss of PINK1, Parkin and DJ-1. We found that intact mitochondria in PINK1-/- and Parkin-/- cells recapitulate all the defects in mitochondrial respiration observed previously in isolated mitochondria from PINK1-/- and Parkin-/- mouse brains, suggesting that these cells are a valid experimental system to study the underlying mechanisms. Our data showed that these defects are not induced by changes in expression levels and activities of all individual complexes composing the electron transport system in PINK1-/- and Parkin-/-. In parallel, we found that loss of DJ-1 does not affect mitochondrial respiration in intact and permeabilized DJ-1-/- MEFs and in isolated mitochondria from the cerebral cortex of DJ-1-/- mice at 3 months or 2 years of age. Interestingly, the mitochondrial transmembrane potential in MEFs cells is reduced in absence of PINK1, Parkin or DJ-1. We first discovered that this reduction is linked to an increase of the opening of the mitochondrial permeability transition pore in PINK1-/-, Parkin-/- and DJ-1-/- cells. Consistent with earlier reports, production of ROS species is increased in DJ-1-/- MEFs. The accumulation of ROS seems linked to the decreased mitochondrial transmembrane potential and to the increased mitochondrial permeability transition pore opening in DJ-17-MEFs, as antioxidant treatment can restore them, whereas oxidative stress inducers have the opposite effects. Interestingly, the specific inhibition of mitochondrial permeability transition pore opening was able to rescue the transmembrane potential and respiration defects in PINK1-/- and Parkin-/- cells. According to our earlier findings in mouse brains, mitochondrial morphology is similar between PINK1-/- and wild-type cells, indicating that the observed mitochondrial functional defects are not due to morphological changes. The intra-mitochondrial calcium concentration measured after FCCP treatment is increased in PINK1-/- and Parkin-/- but not in DJ-1-/- cells. Together, our study suggests that the excessive mitochondrial permeability transition pore opening may underlie the selective reduction of mitochondrial respiration and transmembrane potential as well as an elevation of mitochondrial calcium observed in PINK1-/- and Parkin-/- cells, while increased ROS production seems to be the origin of mitochondrial functional defects observed DJ-1-/-.

Supported by NINDS (R01 NS041779)

